# Effects of As_2_O_3_ nanoparticles on cell growth and apoptosis of NB4 cells

**DOI:** 10.3892/etm.2015.2651

**Published:** 2015-07-23

**Authors:** XIAOYAN DONG, NING MA, MENGMENG LIU, ZILING LIU

**Affiliations:** Department of Hematological Neoplasms, The First Affiliated Hospital of Jilin University, Changchun, Jilin 130021, P.R. China

**Keywords:** arsenic trioxide, nanotechnology, NB4 cells, growth inhibition, apoptosis

## Abstract

The aim of the present study was to explore the preparation of arsenic trioxide (As_2_O_3_) nanoparticles and examine the antitumor effects of these nanoparticles on NB4 cells. As_2_O_3_ nanoparticles were prepared using the sol-gel method and characterized using transmission electron microscopy and energy dispersive spectroscopy. The results indicated that the As_2_O_3_ nanoparticles prepared in the present study were round or elliptical, well dispersed and had an ~40-nm or <10-nm diameter. The antitumor effects of As_2_O_3_ nanoparticles at various concentrations were analyzed by flow cytometry and the MTT assay, and were compared with those of traditional As_2_O_3_ solution. At the same concentration and incubation time (48 h), the survival rate of cells treated with As_2_O_3_ nanoparticles was significantly lower than that of cells treated with the As_2_O_3_ solution. The growth inhibition rate under both treatments was time- and dose-dependent. In addition, at the same concentration and incubation time, the apoptosis rate of the cells treated with As_2_O_3_ nanoparticles was significantly higher than that of the cells treated with the As_2_O_3_ solution. Furthermore, As_2_O_3_ nanoparticles resulted in a greater reduction in the expression of the anti-apoptotic protein B-cell lymphoma 2 compared with the As_2_O_3_ solution. In conclusion, As_2_O_3_ nanoparticles, prepared using the sol-gel method, were found to produce a stronger cytotoxic effect on tumor cells than that produced by the As_2_O_3_ solution, possibly by inhibiting Bcl-2 expression.

## Introduction

Acute promyelocytic leukemia (APL) is a subtype of acute myelogenous leukemia, accounting for 6.2–40.2% of acute myelogenous leukemia cases. APL manifests rapidly, causing serious illness, and is often accompanied by severe bleeding and disseminated intravascular coagulation. In the past, APL was considered to be ‘the most malignant form of acute leukemia’ (1,2). APL cells are relatively sensitive to chemotherapy (CT); however, it has been frequently observed that CT aggravates bleeding disorders, leading to high early-mortality rates. Despite the sensitivity of APL to CT, the median duration of remission ranges between 11 and 25 months, and only 35–45% of the patients are cured by CT alone (3,4).

Arsenic trioxide (As_2_O_3_) is the primary active component in arsenic. Arsenic is a common, naturally occurring substance that exists in organic and inorganic forms. It has been demonstrated that As_2_O_3_ can induce cell differentiation and apoptosis. As_2_O_3_ yields a remission rate of as high as 90% in treating APL (5,6). The traditional As_2_O_3_ solution, however, has numerous side effects, such as hyperleukocytosis, liver and kidney dysfunction, and effusion (7,8). These side effects increase the suffering of patients with APL, which sometimes results in patients decreasing the dose of As_2_O_3_ or even stopping halfway through therapy, thus seriously affecting its curative effects. The aforementioned reasons limit the use of As_2_O_3_ in clinical practice; therefore, developing new methods of As_2_O_3_ administration that avoid these side effects is imperative. Nanomedicine has attracted considerable focus due to its beneficial characteristics, including targeted drug delivery and slow drug release (9). Employing nanotechnology in cancer treatment is currently one of the most cutting-edge fields of biotechnology research (10,11). In the present study, the traditional As_2_O_3_ preparation technology was modified, and As_2_O_3_ nanoparticles were prepared using modern nanotechnology. The aim of the present study was to evaluate the properties of the prepared As_2_O_3_ nanoparticles and investigate their antitumor effects. We hypothesized that the modified preparation technique would improve bioavailability, which would reduce the drug dosage and toxicity and enhance the associated curative effects.

## Materials and methods

### 

#### Cells and cell culture

NB4 cells, a human APL cell line, were provided by Dr Jifan Hu at Stanford University Medical School (Palo Alto, CA, USA) and maintained in the laboratory of The First Affiliated Hospital of Jilin University (Changchun, China). The cells were cultured at 37°C in Iscove's Modified Dulbecco's Medium (Gibco-BRL, Grand Island, NY, USA), supplemented with 10% heat-inactivated fetal bovine serum (Hanzhou Sijiqing Biological Engineering Materials Co., Ltd., Hanzhou, China), 100 U/ml penicillin and 100 µg/ml streptomycin in an atmosphere of 5% CO_2_ and 100% humidity.

#### Preparation of As_2_O_3_ nanoparticles using the sol-gel method

For the preparation method, the following formulae, in which

**Figure:**
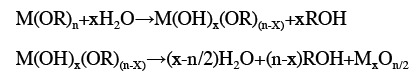


M represents the metal element and R represents C_m_H_2m+1_, were used (12):

The specific preparation method was as follows: All items used in the tests were sterilized, and As_2_O_3_ powder and hydrochloric acid were magnetically stirred and mixed at a mass/volume ratio of 1:0.02-0.1 for 10–30 min. Ethanol was then added at a volume ratio of 1:5-10, the solution was stirred for 20–30 min at 50–60°C and the mixture was sonicated for 5 min. Finally, distilled water was added at a volume ratio of 1:4-5, and the mixture was sonicated for 10–20 min. Following preparation, a few drops of the sample were placed on a copper mesh, dried and then characterized with transmission electron microscopy (TEM; JEM-2010, JEOL Ltd., Tokyo, Japan), scanning electron microscopy (JSM-840, JEOL Ltd.) and energy dispersive spectrometry (EDS; JEOL Ltd.).

#### Cytotoxicity analysis

The cytotoxicity and sensitivity of As_2_O_3_ (Institute for Drug Control of the Ministry of Health of China) and As_2_O_3_ nanoparticles were measured using the MTT cell viability method (13). Cells were divided into three groups: NB4, NB4 + As_2_O_3_ and NB4 + As_2_O_3_ nanoparticles. NB4 cells in the logarithmic growth phase were seeded on 96-well plates in quadruplicate at a density of 1×10^5^/well in 100 µl. As_2_O_3_ solution and As_2_O_3_ nanoparticles (7 different final concentrations: 0.25, 0.5, 1.0, 1.5, 3.0, 6.0 and 12.0 µmol/l) were added at the appropriate time points according to the group setting. After 24, 48, 72 and 96 h of treatment, the cells were incubated for 4 h with MTT (Changchun Biotech Co., Ltd., Changchun, China) and then lysed with acidified isopropanol. Absorbance was measured at 570 nm. The inhibition rate was calculated using the following formula: Inhibition rate = [(absorbance value of control group - absorbance value of test group)/absorbance value of control group] ×100%. All experiments were repeated three times.

#### Flow cytometric analysis

Cell apoptosis was quantified using flow cytometry (FCM; FACSCalibur™, BD Biosciences, San Jose, CA, USA). Cells were grouped as previously described for the cytotoxicity analysis. Both the As_2_O_3_ solution and As_2_O_3_ nanoparticles were tested at two concentrations: 1.5 and 3.0 µmol/l. Following incubation at 37°C in an atmosphere of 5% CO_2_ and 100% humidity for 48 h, the cells were washed with cold phosphate-buffered saline (PBS) twice and resuspended in cold PBS. The apoptosis rate of NB4 cells after treatment was examined using an Annexin V/Propidium Iodide Apoptosis Detection Assay kit (Beyotime Institute of Biotechnology Co., Shanghai, China). All experiments were repeated three times.

#### Western blot analysis

Cells were grouped and pretreated as previously described in the FCM. The total protein was extracted, separated using SDS-PAGE and transferred to a nitrocellulose membrane. The membrane was blocked with 5% skimmed milk at room temperature for 2 h, and then stained with rabbit polyclonal anti-B-cell lymphoma 2 (Bcl-2; 1:200; BA0412) or mouse monoclonal anti-β-actin antibodies (1:200; BM0627; Wuhan Boster Biotechnology Co., Ltd., Wuhan, China) for 2 h at room temperature. Following washing, the membrane was incubated with horseradish peroxidase-labeled goat-anti-rabbit IgG (Sigma-Aldrich, St. Louis, MO, USA) at 1:1,000 for 1 h at room temperature. The membrane was then washed and developed. All experiments were repeated three times.

#### Statistical analysis

All data were analyzed using SPSS 13.0 software (SPSS Inc., Chicago, IL, USA). A *t*-test was adopted to analyze inter-group differences. P<0.05 was considered to indicate a statistically significant difference.

## Results

### 

#### Characteristics of the As_2_O_3_ nanoparticles

**Figure 1.**
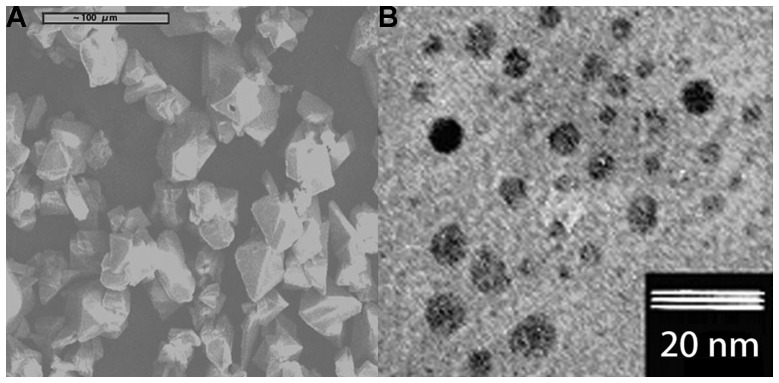
Morphology of As_2_O_3_ powder and nanoparticles. (A) As_2_O_3_ powder under scanning electron microscopy (magnification, ×10,000); (B) As_2_O_3_ nanoparticles (<10 nm) under transmission electron microscopy (magnification, ×300,000). As_2_O_3_, arsenic trioxide.

**Figure 2.**
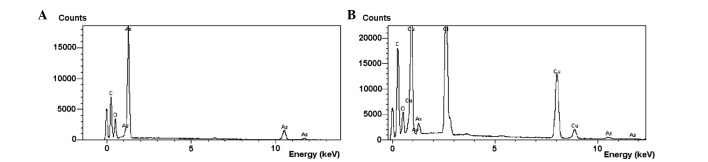
Energy dispersive spectrometry results of (A) As_2_O_3_ powder and (B) As_2_O_3_ nanoparticles. As_2_O_3_, arsenic trioxide.

As observed using TEM, the As_2_O_3_ powder was square, polygonal or in the form of anomalistic crystals with high electron density (Fig. 1A). The average diameter of the As_2_O_3_ powder was >1 µm. By contrast, the As_2_O_3_ nanoparticles were well dispersed, approximately spherical or elliptical and ~40 or <10 nm in diameter (Fig. 1B). The EDS results confirmed that these nanoparticles were As_2_O_3_ (Fig. 2).

#### NB4 cell morphological changes

**Figure 3.**
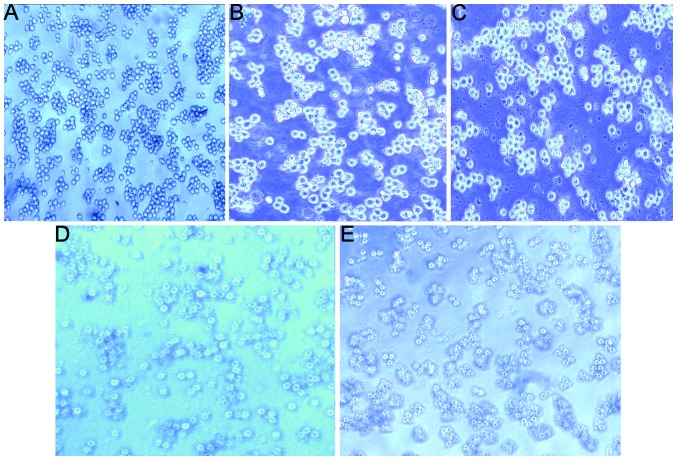
Optical inverted microscopy images of NB4 cells incubated with As_2_O_3_ solution or As_2_O_3_ nanoparticles for 48 h (magnification, ×40). NB4 cells were treated as follows: (A) Control, (B) As_2_O_3_ solution (1.5 µmol/l), (C) As_2_O_3_ nanoparticles (1.5 µmol/l), (D) As_2_O_3_ solution (3.0 µmol/l) and (E) As_2_O_3_ nanoparticles (3.0 µmol/l). As_2_O_3_, arsenic trioxide.

The morphological changes of the NB4 cells were compared following treatment with As_2_O_3_ or As_2_O_3_ nanoparticles, and the results are shown in Fig. 3. The NB4 cells in the control group exhibited a normal shape with similar sizes and clear edges (Fig. 3A). No cell fragmentation was observed (Fig. 3A). Forty-eight hours after As_2_O_3_ solution treatment (1.5 µmol/l), the NB4 cells were found to be reduced in number and volume and to exhibit irregular shapes (Fig. 3B). In addition to the changes described above, the number of necrotic cells and cell fragments increased during incubation at 3.0 µmol/l (Fig. 3D). Using the same concentrations and incubation time, the morphological changes were more marked following treatment with As_2_O_3_ nanoparticles (Fig. 3C and E).

#### As_2_O_3_ nanoparticles inhibit NB4 cell growth

**Figure 4.**
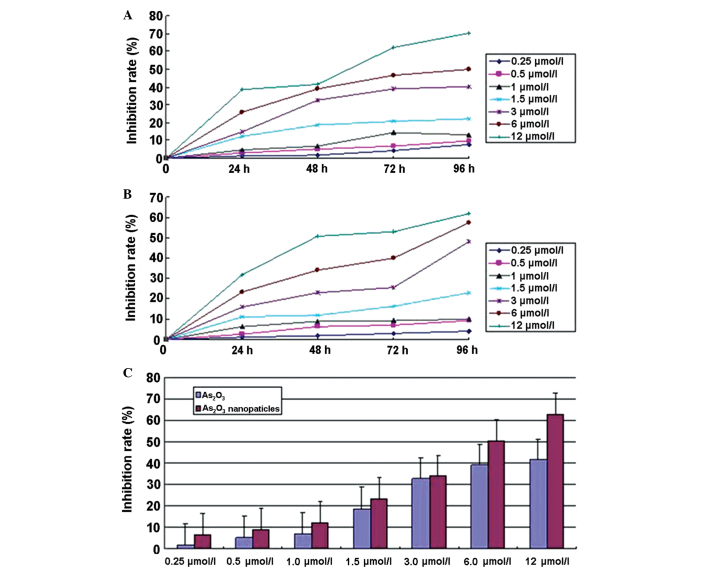
Comparison between the growth-inhibition effects of As_2_O_3_ solution and nanoparticles. The growth-inhibition effects of As_2_O_3_ solution and nanoparticles on NB4 cells were compared at different incubation time-points (24, 48, 72 and 96 h). Growth-inhibitory effects of (A) As_2_O_3_ solution and (B) As_2_O_3_ nanoparticles. (C) Following the treatment of NB4 cells with As_2_O_3_ solution or nanoparticles for 48 h at different concentrations, the growth-inhibitory effects were compared. As_2_O_3_, arsenic trioxide.

The results indicated that the As_2_O_3_ nanoparticles were more effective than the As_2_O_3_ solution in inhibiting NB4 cell growth. At the same concentration and incubation time, the growth rate of cells treated with As_2_O_3_ nanoparticles was significantly lower than that of cells treated with the As_2_O_3_ solution. In both groups, the inhibition rate was time- and dose-dependent (Fig. 4).

#### As_2_O_3_ nanoparticles induce NB4 cell apoptosis

**Figure 5.**
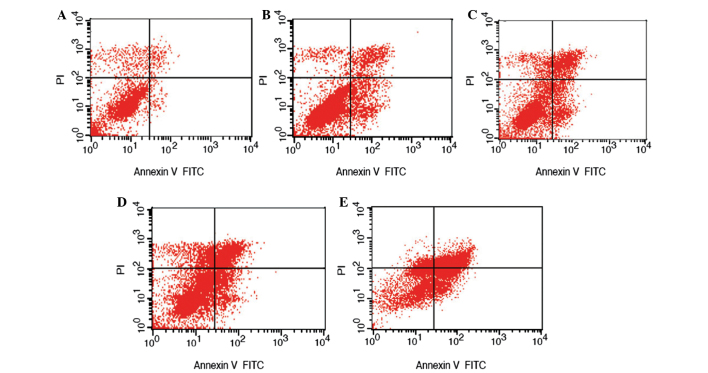
Apoptosis of NB4 cells 48 h after treatment with As_2_O_3_ solution or As_2_O_3_ nanoparticles. NB4 cells were treated as follows (the percentage of apoptotic cells is indicated): (A) Control, 0.86%; (B) As_2_O_3_ solution (1.5 µmol/l), 8.60%; (C) As_2_O_3_ nanoparticles (1.5 µmol/l), 10.44%; (D) As_2_O_3_ solution (3.0 µmol/l), 10.34%; (E) As_2_O_3_ nanoparticles (3.0 µmol/l), 23.41%. As_2_O_3_, arsenic trioxide; PI, propidium iodide; FITC, fluorescein isothiocyanate.

As_2_O_3_ nanoparticles appeared to be more effective than As_2_O_3_ solution in inducing apoptosis of NB4 cells. Using the same concentration and incubation time, the apoptosis level of cells treated with As_2_O_3_ nanoparticles was significantly higher than that of cells treated with the As_2_O_3_ solution (Fig. 5).

#### As_2_O_3_ nanoparticles downregulate Bcl-2 expression

**Figure 6.**
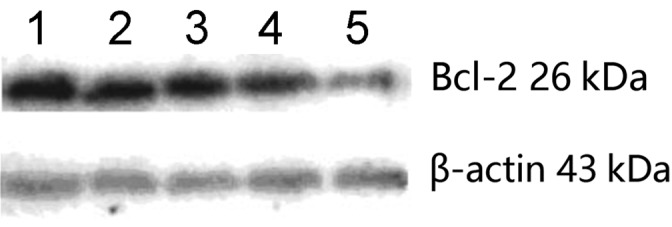
Bcl-2 expression of NB4 cells following treatment with As_2_O_3_ solution or As_2_O_3_ nanoparticles for 48 h. NB4 cells were treated as follows: 1, Control; 2, As_2_O_3_ solution (1.5 µmol/l); 3, As_2_O_3_ nanoparticles (1.5 µmol/l); 4, As_2_O_3_ solution (3.0 µmol/l); 5, As_2_O_3_ nanoparticles (3.0 µmol/l). As_2_O_3_, arsenic trioxide; Bcl-2, B-cell lymphoma 2.

The promotion of apoptosis by As_2_O_3_ was further explored by examining the expression level of the anti-apoptotic protein Bcl-2, which has been implicated in several types of cancer (14). The results showed that, during incubation at 3.0 µmol/l, As_2_O_3_ nanoparticles caused a more significant reduction in Bcl-2 expression compared with the As_2_O_3_ solution (Fig. 6).

## Discussion

As_2_O_3_ is the primary active component in arsenic. In Traditional Chinese Medicine, arsenic has been shown to exhibit excellent efficacy in treating APL (15). As_2_O_3_ acts on APL cells by promoting differentiation, as well as by inhibiting growth and inducing apoptosis. Arsenic acid, however, is a highly toxic substance and its clinical application is therefore limited due to its severe side effects, which include serious heart toxicity, cavity effusion, liver and kidney damage, gastrointestinal adverse reactions and peripheral nervous infection (16,17). It is therefore important to develop new formulations of As_2_O_3_ with high efficiency and low toxicity.

Nanotechnology is a technologically advanced field that manipulates atoms and molecules on a spatial scale ranging from 0.1 to 100 nm in order to serve specific functions. Changing the traditional drug preparation technology by adopting modern nanotechnology improves bioavailability by promoting drug absorption by cells from the tissue space. Nanotechnology also enables slow drug release, increasing drug concentrations in the lesion site, reducing drug dosage and toxicity in non-targeted sites and enhancing the curative effects. The sol-gel method is one of the most common methods for preparing nanomaterials (18).

In the present study, As_2_O_3_ nanoparticles, measuring <10 nm and ~40 nm in diameter, were successfully prepared using the sol-gel method. The results showed that, compared with the As_2_O_3_ solution, the As_2_O_3_ nanoparticles resulted in more significant morphological changes in the NB4 cells (Fig. 3), exhibited stronger growth-inhibition effects in the MTT assay (Fig. 4), induced apoptosis to a greater extent (Fig. 5) and caused greater downregulation of the anti-apoptotic protein Bcl-2 at high concentrations (Fig. 6). These findings were in agreement with a previous report that proposed that As_2_O_3_ induced apoptosis mainly through downregulating the expression of Bcl-2 (19).

The fact that the growth-inhibition and apoptosis-induction functions of As_2_O_3_ nanoparticles are more effective than those of the traditional As_2_O_3_ solution may be due to unique physical and chemical properties, including higher chemical activities; higher absorption and utilization; slow drug release, which helps maintain effective drug concentrations *in vivo*; increased ease of uptake by tumor cells and special pharmacological effects. The surface of nanoparticles can be modified chemically and biologically to generate nano-targeting drugs. In addition to the aforementioned inherent advantages of nanoparticles, nano-targeting drugs ensure targeted drug delivery, increasing curative effects and reducing drug dosages to mitigate or avoid side effects (20). The effectiveness of nanomedicine is directly associated with the size of the nanoparticles. A decrease in particle size causes an increase in surface area and, correspondingly, an increase in the number of surface atoms, leading to higher chemical activities. Small particles, however, are more prone to aggregation, which increases the total size of the particles and offsets the effects of increased chemical activity; therefore, when preparing small nanoparticles, it is necessary to adopt special methods of preventing aggregation. Ultrasonic dispersion is a common and effective method (21,22). In the present study, As_2_O_3_ nanoparticles were prepared using the sol-gel method as previously described (23–24). Unlike in previous studies, smaller-sized As_2_O_3_ nanoparticles (<10 nm) were prepared for the present experiments. The findings showed that small As_2_O_3_ nanoparticles (<10 nm) generate significant antitumor effects, even at low concentrations (1.5 µmol/l), confirming that small As_2_O_3_ nanoparticles may have increased activity, thus requiring reduced dosages for cancer treatment.

In the present study, the traditional preparation technology was modified by employing modern nanotechnology, and smaller As_2_O_3_ nanoparticles were successfully prepared. The growth-inhibition and apoptosis-induction functions of As_2_O_3_ nanoparticles were further characterized, and the superiority of As_2_O_3_ nanoparticles over traditional As_2_O_3_ solutions *in vitro* was demonstrated. In conclusion, As_2_O_3_ nanoparticles are a promising approach for treating APL and the current data provide a theoretical and experimental basis for applying nanomedicine in the clinical treatment of acute leukemia.
